# Improving Meat Quality and Lipid Metabolism of Finishing Pigs by Replacing Dietary Soybean Meal with Enzyme–Bacteria Co-Fermented Rapeseed Meal

**DOI:** 10.3390/foods15030587

**Published:** 2026-02-06

**Authors:** Luobin Yang, An Tao, Hailong Hu, Minfeng Ding, Jun Chen, Xin Li, Xingping Chen, Tiande Zou, Jinming You

**Affiliations:** Jiangxi Province Key Laboratory of Animal Nutrition and Feed, Jiangxi Province Key Innovation Center of Integration in Production and Education for High-Quality and Safe Livestock and Poultry, Jiangxi Agricultural University, Nanchang 330045, China; 19816334350@163.com (L.Y.); junchen@jxau.edu.cn (J.C.); lixin_andy@163.com (X.L.); cxp0315@jxau.edu.cn (X.C.)

**Keywords:** fermented rapeseed meal, growth performance, meat quality, lipid metabolism, finishing pig

## Abstract

This study aimed to investigate the effects of enzyme-bacteria co-fermented rapeseed meal (FRSM) on the growth performance, serum biochemical parameters, meat quality, and lipid metabolism of finishing pigs. A total of twenty-eight Duroc × Landrace × Yorkshire finishing pigs (4 months of age; initial body weight: 60.92 ± 1.08 kg) were randomly allotted to one of four dietary treatments for a 45-day feeding trial, consisting of corn-soybean meal diet (CSD) and three experimental diets in which 50, 75 and 100% of soybean meal in the corn-soybean diet was replaced with FRSM. Results showed that replacing soybean meal with FRSM had no negative effects on the growth performance of finishing pigs, maintaining average daily gain and feed efficiency (*p* > 0.05). Compared with the CSD group, the FRSM group exhibited lower serum cholesterol (*p* < 0.05). The serum low-density lipoprotein cholesterol, alanine aminotransferase, and urea content levels were lower in the FRSM75 or FRSM100 groups than in the CSD group (*p* < 0.05). Compared to the CSD, FRSM feeding increased the pH_24h_ and triglyceride content but significantly decreased the drip loss, shear force and chewiness in longissimus thoracis et lumborum (LTL) muscle (*p* < 0.05). Importantly, compared with the CSD, FRSM feeding significantly lowered the muscle SFA/UFA ratio, increased the PUFA/SFA ratio, and elevated threonine and valine levels (*p* < 0.05). The FRSM100 group exhibited further increases in umami amino acids (AAs), total essential AAs, and total AAs (*p* < 0.05). Morphological analysis indicated that, compared to CSD, the FRSM100 group had a significantly reduced muscle fiber perimeter in the LTL muscle (*p* < 0.05). Moreover, FRSM feeding up-regulated the expression levels of *MyHC I* and *MyHC IIa* and the lipogenic genes *FASN*, *SREBP1*, and *SCD* (*p* < 0.05). These results indicated that compared with rapeseed meal, FRSM exhibited a positive effect on improving the meat quality and lipid metabolism in finishing pigs and can be used as a suitable alternative protein source for soybean meal in finishing pig diets.

## 1. Introduction

Soybean meal is the primary protein source in livestock and poultry diets; however, in recent years, its market price has increased continuously and exhibited substantial volatility, posing significant challenges to the economic efficiency and sustainability of animal production [1]. According to International Monetary Fund commodity price data, global soybean meal prices increased from approximately 350 USD per metric ton in 2016 to around 480 USD per metric ton in 2022–2023, representing a substantial upward trend. Consequently, the identification and development of cost-effective and efficient alternative protein sources have become an urgent priority for the livestock and feed industries. Rapeseed meal (RSM), a major by-product of oil extraction from rapeseed, has attracted considerable attention due to its wide availability, low cost, and relatively high protein content [2]. Nevertheless, the use of RSM in animal diets is constrained by the presence of multiple antinutritional factors, including glucosinolates, tannins, and phytic acid, as well as reduced palatability [3]. Importantly, these limitations are species- and stage-dependent, with monogastric animals (e.g., pigs and poultry) being particularly sensitive. Consequently, the inclusion level of RSM must be strictly limited in practical dietary formulations—for example, to no more than 8% in weaned piglet diets and generally not exceeding 12% in broiler feeds [4].

Over the past decade, microbial fermentation has gained increasing application as a green and bio-friendly processing strategy for improving the nutritional quality of RSM. This approach not only effectively degrades antinutritional factors but also promotes the production of bioactive metabolites with probiotic functions, such as small peptides, organic acids, and phenolic compounds, during the fermentation process, thereby improving feed quality and functional properties [5]. For example, RSM fermented with *Saccharomyces cerevisiae* or *Saccharomyces boulardii* exhibited marked reductions in glucosinolates and 3-butenyl isothiocyanate, while increasing crude protein, minerals (e.g., copper, zinc), and phenolic compounds such as sinapic and gallic acid [6]. However, conventional microbial fermentation is constrained by the limited activity and substrate specificity of enzymes secreted by microorganisms, often resulting in relatively low processing efficiency and incomplete degradation of antinutritional factors. Against this background, enzyme–microbe co-fermentation has emerged as a more effective strategy. This approach involves the supplementation of exogenous enzymes, such as cellulase, glucanase, and phytase, to pre-hydrolyze plant cell walls and macromolecular antinutritional components, thereby alleviating inhibitory effects on microbial growth and providing more readily utilizable substrates for beneficial microorganisms [7,8]. Under these conditions, microbial metabolic activity is enhanced, promoting the synthesis of bioactive components including small peptides and organic acids. The synergistic interaction between exogenous enzymes and microorganisms not only significantly improves processing efficiency but also further optimizes the nutritional quality of the final fermented product [7,8].

Although fermentation technologies have achieved substantial progress in improving the nutritional quality of RSM, the optimal substitution level and maximum application potential of fermented rapeseed meal (FRSM) in finishing pigs remain unclear, particularly with respect to the feasibility of completely replacing soybean meal. This uncertainty stems from conflicting reports. Some studies indicate that high substitution levels (>50%) may reduce feed intake and growth performance, potentially due to residual antinutritional factors or palatability issues [9]. Conversely, other studies have demonstrated that optimized fermentation can fundamentally improve RSM quality, allowing its complete substitution for soybean meal in Nile tilapia, ducks, and sheep while maintaining production performance and improving muscle amino acid and fatty acid composition [10,11,12]. Notably, even in its unfermented state, RSM demonstrates potential as a soybean meal replacer in porcine models. Previous research indicates that replacing soybean meal with a combination of RSM and faba beans did not compromise the growth performance and carcass traits of finishing pigs. Furthermore, it improved meat color (reduced lightness and yellowness) and accumulated free amino acids and sweet-tasting metabolites in muscle, thereby enhancing pork flavor [13]. These findings indicate that RSM has an intrinsic potential to modulate meat quality, and that fermentation processing may further enhance its nutritional value and biological functionality. Moreover, the potential of fermented feeds to enhance meat quality may be linked to the regulation of lipid metabolism. For instance, dietary fermented feed has been shown to increase intramuscular fat (IMF) and improve the amino acid and fatty acid profiles of the longissimus thoracis et lumborum (LTL), effects correlated with the upregulation of lipid metabolism-related genes [14]. Additionally, bioactive compounds inherently present in or generated during the fermentation of agricultural by-products are thought to contribute to improved growth performance and meat quality [15,16].

Despite the promising results of FRSM in various animal models, evidence for its complete substitution of soybean meal in finishing pig diets, particularly regarding meat quality and underlying mechanisms, remains limited. Therefore, we hypothesized that increasing dietary substitution of soybean meal with FRSM would maintain growth performance while improving feed efficiency and meat quality through coordinated effects on nutrient metabolism and muscle characteristics. To test this hypothesis, finishing pigs were offered diets containing 0%, 50%, 75%, or 100% FRSM, and a series of integrated phenotypic, biochemical, and molecular assessments was conducted. Collectively, this work serves as a scientific reference supporting the rational application of FRSM in diets for finishing pigs.

## 2. Materials and Methods

### 2.1. Animal Ethics Statement

Approval for animal experiments was obtained from the Animal Care and Use Committee at Jiangxi Agricultural University (Nanchang, China, approval number: JXAULL-2025-02-08). The feeding trial took place from March to April 2025.

### 2.2. Preparation of FRSM via Microbial-Enzymatic Synergy

A fermentation substrate was prepared by thoroughly mixing RSM and soybean meal at a 9:1 ratio (*w*/*w*) in a blender. The following were sequentially added: 7.5% wheat bran, 4% molasses, and 0.45% alkaline protease. Subsequently, water (110% of the substrate dry weight) along with a 5% inoculum of *Saccharomyces cerevisiae* and a 5% inoculum of *Bacillus subtilis* were sprayed into the mixture to achieve an initial solid-to-liquid ratio of 1:1.20 (kg/L). After homogenization, the material was placed in fermentation bags fitted with one-way valves and subjected to aerobic fermentation at 37 °C for 24 h. This was followed by spraying with a 5% inoculum of *Lactobacillus plantarum*, which adjusted the final solid-to-liquid ratio to 1:1.25 (kg/L), and then switching to anaerobic fermentation at 37 °C for an additional 36 h, resulting in a total fermentation time of 60 h. Upon completion, the fermented product was dried, ground, and stored for subsequent use. All microbial strains were sourced from the China Center of Industrial Culture Collection. The RSM, soybean meal, and wheat bran were purchased from Gao’an Huada Animal Husbandry Co., Ltd. (Yichun, Jiangxi, China). The alkaline protease was sourced from Beijing Solarbio Science & Technology Co., Ltd. (Beijing, China). The chemical composition of RSM mixture before and after fermentation are available in Supplementary Table S1.

### 2.3. Experimental Design and Experimental Diets

A total of twenty-eight Duroc × Landrace × Yorkshire (DLY) barrows with an initial body weight of 60.92 ± 1.08 kg were randomly assigned to one of four dietary treatments consisting of corn-soybean meal diet (CSD), and three experimental diets in which 50, 75 and 100% of soybean meal in the corn-soybean diet was replaced with FRSM. All diets were designed to comply with the nutrient recommendations of the NRC (2012) [17] for finishing pigs. Details of diet formulation and nutrient composition are reported in Table 1. All pigs were allowed a 7-day acclimatization period before the feeding trial. The formal feeding trial was conducted for 45 days, with feed and water available ad libitum.

### 2.4. Sample Collection and Carcass Characteristics Measurements

Feed was withdrawn for 12 h before slaughter in all pigs. Approximately 15 mL of blood was drawn from the anterior vena cava, followed by centrifugation at 3500× *g* for 15 min at 4 °C to obtain serum. Slaughter procedures were conducted in accordance with the *Good Practice of Animal Slaughter—Pig* (GB/T 19479-2019 [18], China). Pigs were slaughtered on-site without pre-slaughter transport, electrically stunned at 110 V, and exsanguinated. Following slaughter, the carcass was divided along the dorsal midline. The heart, liver, spleen, lungs, and kidneys were excised and individually weighed. Carcass traits, including carcass weight, carcass length, average backfat thickness, and loin eye area, were determined strictly in accordance with the *Technical Specification for Measurement of Carcass Traits in Lean-Type Pigs* (NY/T 825-2004 [19], China). After slaughter, LTL tissue was sampled from the left carcass side within the rib region spanning the 10th to 13th ribs. No carcass aging process was applied. Portions of the muscle samples were reserved for the determination of meat quality traits. For subsequent analyses of amino acid composition, fatty acid profiles, and gene expression, the remaining muscle samples were snap-frozen in liquid nitrogen and stored at −80 °C.

### 2.5. Growth Performance Measurements

All pigs were weighed after fasting on the morning of the trial’s commencement and conclusion to obtain initial and final weights, from which the average daily gain (ADG) was calculated. Daily feed consumption was calculated from the difference between feed supplied and residual feed for individual pigs. Average daily feed intake (ADFI) was obtained from the recorded data, whereas feed efficiency (F/G) was defined as the ADFI-to-ADG ratio.

### 2.6. Serum Biochemical Measurements

Serum biochemical indices, including total protein (TP), globulin (GLO), glucose (GLU), triglycerides (TG), urea (UREA), high-density lipoprotein cholesterol (HDL-C), total cholesterol (TC), low-density lipoprotein cholesterol (LDL-C), aspartate aminotransferase (AST), albumin (ALB), and alanine aminotransferase (ALT), were measured on an automated biochemical analyzer (BS-420; Mindray, Shenzhen, China) together with corresponding commercial reagent kits.

### 2.7. Meat Quality Measurements

Meat quality indicators were measured based on the protocol of Lu et al. [20], with slight adaptations. The detailed procedures were as follows: At 45 min and 24 h post-slaughter, the pH of the LTL was measured in different regions using a pH meter (Hanna Instruments, Padua, Italy). At the same time points, meat color parameters, including lightness (L*), redness (a*), and yellowness (b*), were assessed with a 3nh colorimeter (China), and the mean of three replicate measurements was used for analysis. Within 2 h post-slaughter, a portion of LTL tissue was weighed (recorded as M1), transferred into a drip loss tube, and suspended at 4 °C for 24 h. After carefully blotting the surface moisture, the sample was reweighed (recorded as M2). The drip loss percentage was calculated using the following formula: Drip loss (%) = [(M1 − M2)/M1] × 100. The cooking loss of the LTL was determined after 24 h of storage at 4 °C. Briefly, a 2.5 cm-thick LTL sample was blot-dried, weighed (recorded as M3), vacuum-sealed, and heated in a water bath at 80 °C until the core temperature reached 75 °C. After cooling and blotting dry, the sample was reweighed (M4). The cooking loss percentage was calculated as: Cooking loss (%) = [(M3 − M4)/M3] × 100. The cooked samples were subsequently used for texture profile analysis (TPA). Samples were cut into 1.0 cm–thick slices, and textural parameters, including cohesiveness, springiness, gumminess, chewiness, and hardness, were evaluated with a CT3 texture analyzer (Brookfield, Middleboro, MA, USA) based on three replicate measurements per sample. Shear force, representing tenderness, was measured according to the method of Yusuf et al. with minor modifications [11]. Briefly, cooked and cooled meat samples were cut into 25 mm × 25 mm cubes parallel to the muscle fiber orientation. Shear force was measured perpendicular to the fiber direction using a shear device. The value for each sample, expressed in Newtons (N), was obtained by averaging three replicates. Furthermore, muscle moisture and IMF contents were determined following standardized procedures for pork quality evaluation (NY/T 821-2019 [21], China). Commercial assay kits from Nanjing Jiancheng Bioengineering Institute (Nanjing, China) were used to quantify TG and TC in the LTL.

### 2.8. Amino Acid Analyses

Liquid nitrogen-ground muscle powder (100 mg) was weighed into a centrifuge tube, followed by the addition of 1000 μL of extraction solvent (methanol: acetonitrile: water = 2:2:1, *v*/*v*) and vortex mixing. The mixture was then subjected to ice-water bath ultrasonication for 10 min and immediately snap-frozen in liquid nitrogen for 1 min. This freeze–thaw procedure was performed for three cycles. Following completion, the samples underwent storage at −20 °C for 1 h and subsequent centrifugation (13,000 rpm, 15 min, 4 °C). The collected supernatant was then dried under nitrogen stream, and the resulting residue was reconstituted in 100 μL of acetonitrile–water (1:1, *v*/*v*), vortex-mixed for 30 s, and sonicated in an ice-water bath for 10 min. A final centrifugation (13,000 rpm, 4 °C, 15 min) was performed, after which the supernatant was subjected to LC-MS analysis.

Liquid chromatography parameters: Data acquisition was achieved via a UHPLC system (ACQUITY UPLC, Waters, Milford, MA, USA) interfaced with an AB SCIEX 6500 QTRAP mass spectrometer (AB SCIEX, Concord, ON, Canada). Separation utilized a Waters ACQUITY UPLC BEH Amide column (Waters, Milford, MA, USA) at 25 °C. The mobile phase was prepared as follows: (A) 25 mM ammonium hydroxide and 25 mM ammonium acetate in water; (B) acetonitrile. For both positive and negative ionization modes, the same compositions of phase A and B were employed. Chromatographic separation was performed at a flow rate of 0.3 mL/min. Gradient elution was applied as follows: 95% B from 0 to 1 min, decreased to 65% B over 1–14 min, further reduced to 40% B between 14 and 16 min, maintained at 40% B from 16 to 18 min, returned to 95% B from 18 to 18.1 min, and held at 95% B until 23 min. Samples were injected at a volume of 2 μL. During data acquisition, the sample injection order was randomized.

Mass spectrometry parameters: The ESI source parameters were set as follows: nebulizing gas (Gas 1), 60 psi; auxiliary heating gas (Gas 2), 60 psi; curtain gas (CUR), 30 psi; ion source temperature, 600 °C; and ion spray voltage (ISVF), ±5500 V (for both positive and negative modes). Data acquisition was conducted on a triple quadrupole mass spectrometer operated in MRM mode, using nitrogen as the collision gas at a medium setting. The declustering potential and collision energy for individual MRM transitions were optimized separately. Data were acquired in scheduled MRM (sMRM) mode.

### 2.9. Fatty Acid Composition Analyses

Muscle tissues were cryogenically pulverized with liquid nitrogen. An accurately weighed 100 mg portion of the resulting powder was placed in a centrifuge tube and extracted by stepwise addition of pre-chilled methanol (300 μL), methyl tert-butyl ether (1 mL), and a fatty acid internal standard (2 μL, 1 mg/mL). After brief vortex mixing (30 s), the samples were subjected to low-temperature ultrasonication for 15 min. Thereafter, 300 μL of pure water was added, and the mixture was homogenized and allowed to stand at 4 °C for 10 min. The sample was centrifuged at 4 °C and 15,800× *g* for 10 min, and the upper ether phase was collected into a new centrifuge tube. The sample was dried under a nitrogen stream and stored at −80 °C until further use. Prior to instrumental analysis, the sample was taken out from the −80 °C freezer and placed on ice. It was reconstituted with 200 μL of pre-cooled acetonitrile/isopropanol/water (65:30:5, *v*/*v*/*v*) solution, vortexed for 1 min, and ultrasonicated at low temperature for 15 min. After centrifugation (15,800× *g*, 4 °C, 30 min), the supernatant was membrane-filtered (0.22 μm) into a vial. For quality control, equal volumes of supernatant from each sample were pooled to prepare a QC sample, which was then subjected to LC–MS analysis.

Chromatographic conditions: Separation was performed on a Waters ACQUITY UPLC BEH C18 column (2.1 × 100 mm, 1.7 μm; Waters, USA) maintained at 55 °C. The injection volume was 5 μL. The mobile phase consisted of (A) 40% H_2_O + 60% acetonitrile + 10 mM ammonium acetate and (B) 90% isopropanol + 10% acetonitrile + 10 mM ammonium acetate. The gradient elution program was set as follows: 0–0.75 min, 32% B; 0.75–7.75 min, 85% B; 7.75–7.80 min, 97% B; 7.80–9.00 min, 97% B; 9.00–9.05 min, 32% B; 9.05–10.00 min, 32% B. Chromatographic separation was performed at a flow rate of 0.26 mL/min.

Using an electrospray ionization source at 600 °C, mass spectrometric analysis was performed. For positive and negative modes, ion spray voltages of 5500 V and −4500 V were employed, respectively. Curtain gas pressure was maintained at 20 psi, while both nebulizing and auxiliary gases were supplied at 60 psi. The multiple reaction monitoring mode was employed for data acquisition.

### 2.10. Histological and Morphometric Analysis of LTL

LTL samples were fixed in 4% paraformaldehyde, followed by dehydration, embedding, sectioning, hematoxylin and eosin (HE) staining, and sealing, as described by Wang et al. [22]. Subsequently, morphological changes in muscle fibers were observed under a microscope, and fiber diameter, perimeter, cross-sectional area, and density were measured using Image-Pro Plus 6.0 (Media Cybernetics, Rockville, MD, USA), with 35 random images captured per sample.

### 2.11. RNA Extraction and Quantitative Real-Time PCR Analysis

Total RNA was isolated from the LTL with a commercial RNA extraction kit (Accurate Biology, Changsha, China). The concentration and purity of the RNA were determined using a DU800 spectrophotometer (Beckman Coulter, Fullerton, CA, USA). Subsequently, cDNA was synthesized from the extracted RNA with a commercial reverse transcription kit (Accurate Biology, Changsha, China) and stored at −20 °C until further analysis. Quantification of target gene transcripts was performed by real-time PCR with SYBR Green I chemistry, using gene-specific primers summarized in Table 2. β-Actin served as the reference gene, and relative transcript abundance was determined according to the 2^−ΔΔCT^ approach [23].

### 2.12. Statistical Analyses

Informed by empirical precedent in piglets and finishing pigs research [24,25,26], a sample size of seven animals per treatment group was selected. The sufficiency of this sample size was subsequently verified via a post hoc power analysis conducted in G*Power 3.1 (Heinrich Heine University, Düsseldorf, Germany), which confirmed an 80% probability (power) of detecting biologically relevant effect sizes at a significance level (α) of 0.05. All datasets were assessed for normality using the Shapiro–Wilk test and were found to be normally distributed. Subsequently, the data were analyzed by one-way analysis of variance (ANOVA) using SPSS 20.0 (Chicago, IL, USA). When a significant treatment effect was detected, differences among means were assessed using Duncan’s multiple range test. Duncan’s test was selected to facilitate pairwise comparisons among multiple dietary treatments with similar sample sizes. Statistical significance was set at *p* < 0.05, with values of 0.05 < *p* < 0.10 indicating a trend toward significance. Data are expressed as the mean and standard error of the mean (SEM).

## 3. Results

### 3.1. Growth Performance

Ln Table 3, no significant differences (*p* > 0.05) were observed among the four experimental groups for growth performance. Compared with the CSD group, the F/G was reduced by 2.99%, 3.98%, and 1.99% in all FRSM groups, respectively. Overall, the data support that the use of FRSM as an alternative protein ingredient had no detrimental impact on growth-related responses in finishing pigs.

### 3.2. Carcass Characteristics and Viscera Index

Table 4 summarizes the effects of substituting soybean meal with FRSM on carcass characteristics in finishing pigs. Across all treatments, carcass length, carcass weight, carcass yield, loin eye area, and skin thickness remained comparable (*p* > 0.05). The FRSM100 group showed a tendency toward lower suet weight (*p* = 0.063) and average backfat thickness (*p* = 0.064) than the CSD group. Additionally, heart, liver, spleen, kidney, and lung indices did not differ significantly (*p* > 0.05).

### 3.3. Serum Biochemical Indicators

According to Table 5, serum TC content was significantly decreased in all FRSM groups relative to the CSD group (*p* < 0.05). Both the FRSM75 and FRSM100 groups exhibited a significant reduction in serum LDL-C and ALT levels compared to the CSD group (*p* < 0.05). Furthermore, the FRSM100 group showed a highly significant decrease in serum UREA content relative to the CSD, FRSM50, and FRSM75 groups (*p* < 0.01). No significant differences were observed among the FRSM groups for serum TP, ALB, GLO, HDL-C, TG, GLU, or AST levels (*p* > 0.05).

### 3.4. Meat Quality

In Table 6, relative to the CSD group, FRSM100 supplementation resulted in higher pH_24h_ and lower drip loss in the LTL (*p* < 0.05). The shear force of the LTL was significantly lower in the FRSM75 and FRSM100 groups (*p* < 0.05) than that in CSD group. All FRSM groups showed a trend toward higher a_24h_ (*p* = 0.068) and IMF content (*p* = 0.075) than the CSD group. Furthermore, muscle TG levels were increased in pigs receiving FRSM75 and FRSM100 compared with those in the CSD group (*p* < 0.05). Other measured meat quality traits remained unaltered (*p* > 0.05). In summary, FRSM not only had no negative impact on pork quality but also improved it in certain aspects.

### 3.5. Textural Properties of the LTL

With respect to LTL textural characteristics in finishing pigs (Table 7), chewiness was reduced in the FRSM75 and FRSM100 treatments relative to the CSD (*p* < 0.05), whereas the remaining texture attributes were comparable across groups (*p* > 0.05).

### 3.6. Analysis of Free Amino Acids in the LTL

As presented in Table 8, alterations in LTL free amino acid profiles were observed following dietary FRSM inclusion. Relative to the CSD group, threonine and valine contents in the LTL were significantly elevated across all FRSM treatments (*p* < 0.05). Furthermore, the methionine and total amino acid (TAA) contents were significantly higher in the FRSM75 and FRSM100 groups than in the CSD group (*p* < 0.05). Notably, the FRSM100 group demonstrated significantly greater contents of essential amino acids (EAAs) and umami AA than the CSD and FRSM50 groups (*p* < 0.05). Additionally, the methionine and Valine contents in the FRSM100 group were significantly elevated compared to the FRSM50 group (*p* < 0.05). Finally, the contents of isoleucine, leucine, and asparagine in the FRSM100 group were significantly higher than those in the other three groups (*p* < 0.05).

### 3.7. Fatty Acid Analysis of the LTL

As shown in Table 9, dietary replacement of soybean meal with FRSM significantly altered the fatty acid profile in the LTL of finishing pigs. At the overall level, all FRSM groups showed a significantly lower SFA/UFA ratio and a significantly higher PUFA/SFA ratio than the CSD group (*p* < 0.05). Specifically, the contents of C16:2, C18:5, and C21:2 were significantly increased in the FRSM100 group (*p* < 0.05), which also showed an overall increasing trend in the contents of MUFA, PUFA, and UFA. The contents of C20:1 and C21:5 were significantly higher in both the FRSM75 and FRSM100 groups than in the CSD group (*p* < 0.05). Meanwhile, the C18:4 content was significantly elevated across all FRSM groups, whereas C18:0 was significantly reduced only in the FRSM50 group (*p* < 0.05). For the other fatty acids, the measured values did not differ significantly. Collectively, substituting soybean meal with FRSM, especially at a high inclusion level (100%), promotes a healthier unsaturated fatty acid profile in pork.

### 3.8. Analysis of Muscle Fiber Morphology and mRNA Expression of Myofiber-Related Genes in LTL

**Figure 1:**
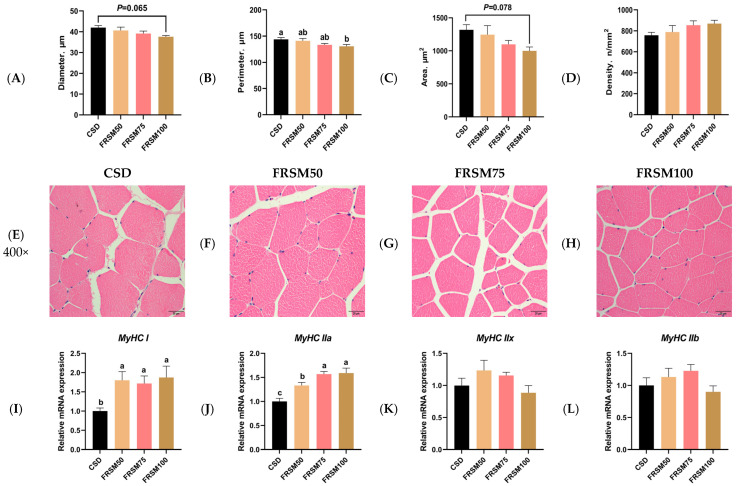
Effect of FRSM replacing soybean meal on both the morphological characteristics and myofiber-related gene expression in the LTL of finishing pigs. (**A**) Muscle fiber diameter. (**B**) Muscle fiber perimeter. (**C**) Muscle fiber area. (**D**) Muscle fiber density. (**E**–**H**) Representative image of H&E-stained muscle fiber morphology in a transverse section of the LTL. Scar bar = 20 μm (400×). (**I**–**L**) Show the mRNA expression levels of myosin heavy chain isoforms (*MyHC I*, *MyHC IIa*, *MyHC IIx*, and *MyHC IIb*) analyzed by real-time quantitative PCR. ^a–c^ Within a row, mean values with different superscripts differ significantly at *p* < 0.05. n = 7.

To investigate morphological changes in muscle fibers, transverse sections of the LTL were prepared and stained with hematoxylin and eosin (H&E). Quality control confirmed uniform staining with clearly discernible muscle fiber structures. The sections were examined under a fluorescence microscope, with randomly selected fields captured for analysis. As shown in Figure 1A–D, compared to the CSD, the FRSM100 group showed a significantly smaller muscle fiber perimeter in the LTL (*p* < 0.05), with decreasing trends also noted in fiber diameter and cross-sectional area. Muscle fiber density did not differ significantly among groups (*p* > 0.05).

Given the close association between muscle fiber morphology and type, we evaluated the expression levels of myofiber type-related genes. As shown in Figure 1I–L, dietary FRSM supplementation increased *MyHC I* and *MyHC IIa* transcript abundance in the LTL relative to the CSD group (*p* < 0.05), whereas *MyHC IIx* and *MyHC IIb* expression remained comparable across treatments (*p* > 0.05).

### 3.9. mRNA Expression of Lipid Metabolism Genes in LTL

**Figure 2:**
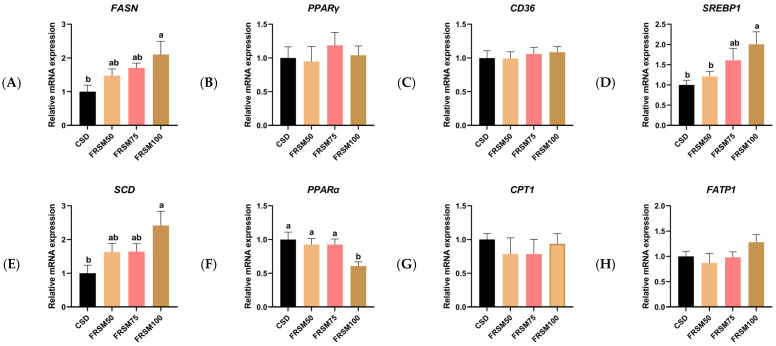
Effect of FRSM replacing soybean meal on expression of lipid metabolism-related genes in the LTL of finishing pigs. (**A**) *FASN*. (**B**) *PPARγ*. (**C**) *CD36*. (**D**) *SREBP1*. (**E**) *SCD*. (**F**) *PPARα*. (**G**) *CPT1*. (**H**) *FATP1*. a, b Within a row, mean values with different superscripts differ significantly at *p* < 0.05. n = 7.

The mRNA expression levels of lipid metabolism-related genes in the LTL are presented in Figure 2. Relative to the CSD, the FRSM100 treatment up-regulated the mRNA expression of *FASN*, *SREBP1*, and *SCD*, while down-regulating that of *PPARα* (*p* < 0.05). In addition, *SREBP1* mRNA expression was higher in the FRSM100 group than in the FRSM50 group (*p* < 0.05). However, *PPARγ*, *CD36*, *CPT1*, and *FATP1* showed no significant differences in expression among the groups (*p* > 0.05).

## 4. Discussion

This study comprehensively evaluated the effects of replacing soybean meal with FRSM in finishing pig diets. The findings demonstrate that high-level substitution, up to 100%, is feasible without compromising growth performance. More importantly, FRSM inclusion conferred significant dual benefits: it improved systemic metabolic health, as reflected in key serum biomarkers, and comprehensively enhanced meat quality. The latter encompassed improvements in physical attributes (such as water-holding capacity and tenderness), nutritional value (amino acid and fatty acid profile), and the potential for flavor development. The underlying mechanisms appear to involve favorable modifications in muscle fiber characteristics and lipid metabolism regulation. These key aspects are discussed below, highlighting how FRSM contributes to producing pork with superior quality and metabolic benefits.

Enhancing growth performance is fundamental in commercial pig farming, while optimizing the F/G ratio is the critical factor directly determining profitability. RSM, as a potential low-cost protein resource, has long been limited in its feeding value due to antinutritional factors such as glucosinolates [27]. Fermentation processing can effectively degrade these antinutritional factors and improve the availability of protein and amino acids. In this study, replacing 50%, 75%, and 100% of soybean meal in the diet with FRSM did not adversely affect the growth performance of finishing pigs. Moreover, the F/G values in all FRSM groups were numerically lower than those in the CSD group, demonstrating a clear economic advantage. Notably, FRSM75 achieved the most favorable F/G ratio, while excessive FRSM inclusion in the FRSM100 diet may have reduced palatability and ADFI, thereby limiting feed efficiency. This result aligns with previous research; for example, adding 12.24% FRSM to replace 35% of crude protein in growing pig diets significantly reduced ADFI while causing no significant differences in average daily gain and F/G [28]. However, an increased F/G was observed when growing pigs were fed a diet with 10% FRSM replacing soybean meal [29]. These discrepancies may arise from variations in fermentation processes, replacement levels, or animal growth stages. Overall, this study achieved a high-level replacement of soybean meal with FRSM during the finishing phase while consistently improving F/G, indicating its promising application potential in enhancing feed conversion efficiency and reducing production costs.

We further analyzed the effects of FRSM on carcass traits and serum metabolic parameters in finishing pigs. Consistent with previous reports that supplemental feeding of fermented complete feed reduced the average backfat thickness of finishing pigs [30], the present study also showed a decreasing trend in backfat thickness and leaf fat weight in the FRSM groups. These changes suggest that FRSM feeding may influence lipid deposition in subcutaneous adipose tissue and be associated with reduced fat accretion. In parallel, significantly lower serum TC and LDL-C levels were observed in the FRSM groups, reflecting a more favorable systemic lipid metabolic status. Furthermore, serum ALT levels decreased significantly with increasing FRSM substitution, demonstrating that even at high replacement levels of soybean meal, FRSM did not impose a burden on liver function but instead contributed to maintaining hepatic metabolic homeostasis. This aligns with reports in weaned piglets, where feeding FRSM significantly reduced plasma ALT activity [31], further supporting a potential hepatoprotective effect of FRSM. Previous studies suggest that FRSM may influence systemic lipid metabolism through multiple pathways, including enhanced lipid digestion, reduced intestinal cholesterol absorption and esterification, and increased cholesterol excretion [32], which may partially explain the observed changes in serum lipid profiles. Serum UREA level is an important indicator for evaluating protein metabolism and nitrogen utilization efficiency [33]. In our study, it was significantly reduced in FRSM100, implying that complete replacement of soybean meal with FRSM helps optimize amino acid balance and improve nitrogen utilization efficiency. This result is supported by previous research in poultry [34]. Overall, these results indicate that FRSM can serve as a soybean meal substitute in finishing pig diets without adverse effects on carcass traits or key serum metabolic indicators.

Currently, improving pork quality can meet the growing consumer demand for high-quality and healthy meat products [35]. Meat quality evaluation is primarily based on indicators including meat color, marbling, water-holding capacity, flavor, as well as nutritional composition [36]. In this study, complete replacement of soybean meal with FRSM resulted in an overall improvement in muscle water-holding capacity and postmortem physicochemical properties in finishing pigs, indicating enhanced meat quality stability and a potentially reduced risk of PSE pork. Both pH and water-holding capacity jointly determine the stability of meat color, and improved water retention can reduce the loss of water-soluble pigments such as myoglobin, resulting in a more vivid and stable meat color [37,38]. This aligns with findings in broilers demonstrating that FRSM supplementation significantly increases breast muscle pH_24h_ and improves thigh muscle water-holding capacity [39]. Muscle tenderness is a key attribute of eating quality, typically measured by shear force, and an increase in IMF content is often associated with reduced shear force [40]. In our study, the overall improvement in tenderness-related traits of the LTL in finishing pigs fed FRSM may be closely associated with enhanced IMF deposition and reduced shear force. This finding is consistent with Liu et al. [14], who showed that fermented feed markedly decreased LTL shear force and increased IMF content in finishing pigs, resulting in improved tenderness.

Furthermore, the morphological characteristics and fiber type composition of muscle fibers are important factors influencing meat quality, particularly tenderness and water-holding capacity [41]. In this study, FRSM supplementation was associated with finer muscle fiber morphology and increased mRNA expression of oxidative fiber–related myosin heavy chain isoforms. These coordinated changes are consistent with muscle characteristics commonly linked to a more oxidative metabolic profile. Previous studies have shown that refined muscle fiber morphology is generally associated with lower shear force and improved tenderness [42]. Moreover, an increased proportion of oxidative fibers is not only positively correlated with enhanced tenderness but also improves muscle metabolic stability and water retention capacity. This provides a histological basis for the observed reduction in drip loss and improvement in water-holding capacity [41,43]. These results are consistent with previous research indicating that fermented feed can modulate muscle fiber type transformation and thereby improve meat quality [14], and further suggest that this process may be governed by specific microorganisms and their metabolites present in the fermented feed. In summary, feeding FRSM was associated with improvements in the water-holding capacity and tenderness of pork. These effects may be related to increased IMF deposition, refined muscle fiber morphology, and a tendency toward more oxidative muscle fiber types. However, whether FRSM contains unknown active factors or specific microbial metabolites capable of regulating these processes requires further investigation.

The amino acid profile of pork is a central indicator for assessing its nutritional value and flavor quality [44]. The composition, content, and proportion of essential amino acids in muscle directly determine the quality of muscle protein [45]. Free amino acids, including umami AAs such as glutamate and aspartate, as well as sweet-tasting amino acids like alanine, glycine, and serine, serve as important flavoring substances and are also key precursors for aroma formation during heating processes [46]. In this study, FRSM feeding was associated with an overall improvement in the muscle amino acid profile of finishing pigs, particularly reflected by increased levels of TAA and EAA. It is worth noting that the contents of umami AA, including glutamate and aspartate, showed a gradual upward trend with increasing FRSM inclusion levels, reaching their highest concentration in the complete replacement group. Similar dose-dependent increases in muscle amino acid content have been reported in broilers fed diets in which soybean meal was progressively replaced by doubly fermented soybean meal, an effect attributed to improved protein digestibility and amino acid utilization [47]. Integrating the above findings, FRSM feeding was associated with an overall increase in TAA and EAA in muscle, as well as an enrichment of umami-related amino acids. These changes may reflect, at least in part, an improvement in protein utilization induced by fermentation (for example, through an increase in small peptides), which could facilitate amino acid digestion, absorption, and subsequent deposition in muscle tissue. Nevertheless, the underlying mechanisms remain unclear, and the observed changes also may involve multiple factors, including alterations in muscle amino acid metabolism or indirect effects of fermentation-derived compounds. Overall, the modified amino acid profile suggests a potential relevance to the nutritional value and flavor-related attributes of pork.

IMF constitutes an important lipid fraction affecting meat flavor and is predominantly present in the form of TG [48]. The extent of IMF deposition and its fatty acid profile jointly affect muscle nutritional quality and oxidative stability, thereby shaping meat quality traits and consumer perception [49]. Data from this study show that the TG content in the LTL of finishing pigs in the FRSM75 and FRSM100 groups significantly increased, suggesting an alteration in IMF deposition levels. This change may be accompanied by alterations in muscle fatty acid composition. Further analysis of fatty acid composition revealed that dietary replacement of soybean meal with FRSM resulted in a reduced SFA/UFA ratio and an increased PUFA/SFA ratio in muscle, indicating a shift toward a more nutritionally favorable lipid profile. From a human nutrition perspective, lower dietary intake of saturated fatty acids and a higher proportion of unsaturated fatty acids are generally associated with improved lipid metabolism and reduced risk of metabolic disorders [50]. Accordingly, pork with a reduced SFA/UFA ratio and elevated PUFA/SFA ratio is often regarded as having enhanced nutritional value and a more positive health perception among consumers [51]. While the contents of both MUFAs and PUFAs showed an increasing trend, with the most pronounced improvement observed in the 100% replacement group. This dose-dependent improvement may be associated with the overall enhancement of available nutrient supply in the diet as the inclusion level of fermented feed increases, a phenomenon also reported in previous studies [14]. Furthermore, these favorable changes in the fatty acid profile, which improve meat quality and nutritional value, are in line with those reported in earlier studies involving ruminants and poultry [11,39]. The enrichment of MUFAs and PUFAs may also contribute to sensory attributes of pork, as unsaturated fatty acids are closely linked to flavor development and mouthfeel. However, it should be noted that although compositional changes suggest potential improvements in nutritional and sensory quality, direct sensory evaluation was not conducted in this study. Therefore, the consumer-perceived impact of these changes remains to be validated in future studies.

These phenotypic alterations described above may stem from coordinated regulatory changes affecting lipid metabolic pathways at the gene level. Gene expression analysis revealed that FRSM supplementation was associated with elevated mRNA expression of key lipogenic regulators, including *SREBP1* and its downstream genes *FASN* (for synthesis) and *SCD* (for desaturation). Among these, *SCD*, as a rate-limiting enzyme involved in the conversion of SFA to MUFA, showed increased expression that was closely associated with higher contents of flavor-and health-related MUFAs in muscle [52,53]. Meanwhile, the expression of *PPARα*, a key regulator of fatty acid oxidation, was suppressed in the FRSM100 group, which may be associated with a reduced capacity for fatty acid oxidation and a relative retention of PUFAs [54]. Collectively, these coordinated changes in gene expression suggest a metabolic profile characterized by enhanced lipogenic potential and attenuated oxidative capacity, which may contribute to IMF deposition and the observed improvement in fatty acid composition. Nevertheless, the effects of FRSM on pork fatty acid composition have received limited scrutiny, and the regulatory mechanisms governing muscle lipid metabolism–related gene expression in finishing pigs fed FRSM are not yet fully understood. Although fermentation clearly altered the chemical composition of RSM, the relative contribution of individual fermentation-induced changes cannot be distinguished in the present study. However, our data revealed that the most pronounced compositional shift was the enrichment of phenolic compounds and flavonoids. Therefore, one possible explanation is that phenolic compounds and flavonoids enriched during fermentation possess potential biological activities related to lipid metabolism, which may be involved in the observed changes in lipid metabolism–related gene expression and fatty acid composition. This inference aligns with previous findings on the involvement of plant bioactive compounds (like rosmarinic acid) in the regulation of key lipid metabolic pathways [55], although the specific molecular mechanisms warrant further investigation for clarification.

## 5. Conclusions

Overall, under conditions employed in the present experiment, replacing soybean meal with FRSM during the finishing phase (45 days) maintained normal growth performance in pigs. Moreover, FRSM supplementation markedly improved pork quality, as reflected by enhanced IMF deposition and tenderness, a more favorable fatty acid profile characterized by increased MUFA and PUFA proportions, and improved amino acid deposition. These improvements were accompanied by coordinated changes in muscle fiber characteristics, suggesting potential physiological associations underlying the observed phenotypic responses.

It should be noted that these conclusions are limited to finishing pigs, the specific fermentation protocol applied, and the relatively short experimental duration. In addition, sensory evaluation and economic analysis were not conducted. Therefore, future studies involving long-term feeding trials across different growth stages, as well as comprehensive cost–benefit assessments, are needed to further confirm the wider applicability of FRSM in commercial pig production.

## Figures and Tables

**Table 1:** Experimental diet composition and nutrient levels (dry matter basis) %.

Raw Material	Dietary Treatments			
	CSD	FRSM50	FRSM75	FRSM100
Corn	66.71	68.88	71.53	74.18
CP43% Soybean meal	16.00	8.00	4.00	0.00
FRSM	0.00	8.00	12.00	16.00
Wheat bran	12.88	9.55	6.55	3.54
Soybean oil	1.25	1.59	1.48	1.37
L-lysine, 78%	0.43	0.68	0.81	0.94
L-methionine, 98%	0.07	0.16	0.21	0.26
L-threonine, 98%	0.13	0.25	0.32	0.38
L-tryptophan, 98%	0.02	0.07	0.09	0.12
L-isoleucine, 98%	0.00	0.13	0.21	0.28
Valine	0.10	0.15	0.23	0.30
Calcium hydrogen phosphate	0.85	0.93	1.02	1.10
Limestone	0.83	0.88	0.82	0.77
Sodium chloride	0.49	0.50	0.50	0.51
Phytase	0.02	0.02	0.02	0.02
Mineral and vitamin premix ^1^	0.22	0.22	0.22	0.22
Calculated nutrient content				
ME, kcal/kg	3150.00	3150.00	3150.00	3150.00
Calcium	0.60	0.60	0.60	0.60
Total phosphorus	0.50	0.50	0.50	0.50
Lysine	0.89	0.89	0.89	0.89
Methionine + Cysteine	0.49	0.49	0.49	0.49
Threonine	0.55	0.55	0.55	0.55
Analyzed nutrient content				
Dry matter	88.20	88.70	88.60	89.10
Crude protein	14.90	14.50	14.90	14.70
Crude fat	4.10	4.40	4.70	4.60
Crude fiber	3.00	3.30	3.30	3.40
Crude ash	4.00	4.80	4.90	4.60

Note: ^1^ The mineral premix supplied the following per kg of diet: Fe (FeSO_4_·H_2_O), 62.5 mg; Zn (ZnSO_4_·H_2_O), 40 mg; Cu (CuSO_4_·5H_2_O), 5 mg; I (KI), 0.15 mg; Mn (MnSO_4_·H_2_O), 2 mg; Se (Na_2_SeO_3_), 0.15 mg. The vitamin premix supplied the following per kg of diet: Vitamin A, 4500 IU; Vitamin D_3_, 1500 IU; Vitamin E, 12 IU; Vitamin K_3_, 1.5 mg; Vitamin B_1_, 1.5 mg; Vitamin B_2_, 3.75 mg; Vitamin B_6_, 1.8 mg; Vitamin B_12_,0.18 mg; Biotin, 0.75 mg; Pantothenic acid, 7.5 mg; Folic acid, 0.75 mg; Niacin, 15 mg.

**Table 2:** Primer sequences used in this study.

Gene	Primer	Product Size/bp	Accession
*FATP1*	F: TGGATGCCTACTCCAATGCT R: CCAGGAAGATAGCCACCACA	81	XM_021076116.1
*FASN*	F: GGTATACGCCACCATCCTCA R: AAGGTCACACCTTGCTCCTT	66	NM_001099930.1
*PPARα*	F: GAGCCTGAGGAAACCCTTCT R: GCACCAAATGATAGCAGCCA	121	NM_001044526.1
*PPARG*	F: TGGCCATTCGCATCTTTCAG R: ATCTCGTGGACGCCATACTT	145	XM_005669783.3
*CD36*	F: CCTGAGACCCACACAGTCTC R: AGGTGTCGTTCTCTGTTCCA	85	XM_005667693.3
*SCD*	F: TAAACAGTGCTGCCCACCTA R: AGGGAAAGGTGTGGTGGTAG	126	NM_213781.1
*CPT1*	F: CACAAGATTTCGCGGTCAGT R: TTGTAGCCCACCAGGACTTT	72	NM_001129805.2
*SREBP1*	F: GCAAGGCCATCGACTACATC R: AGGTTCTCCTGCTTGAGCTT	64	NM_214157.1
β-actin	F: CCCTGGAGAAGAGCTACGAG R: TAGAGGTCCTTGCGGATGTC	178	XM_003124280.5
*MyHC IIx*	F: GGCCACAGATAGTGCCATTG R: GTCAGCAGAGTTCAGACCCT	193	XM_021066024.1
*MyHC IIa*	F: AACGATGCCATCAGGCTCA R: GGTGGATCTGGGTGTCCTTG	145	NM_214136.1
*MyHC IIb*	F: TGCTGTGTTTGCAAAATGGTCT R: TTACCAGATGAAGACGGTGGC	118	XM_021066035.1
*MyHC I*	F: AGGCCTTTCGACCTCAAGAA R: TGGTCCTCCTTCACAGTCAC	140	NM_213855.2

Note: β-actin = beta-actin; *MyHC I* = myosin heavy chain I; *MyHC IIa* = myosin heavy chain IIa; *MyHC IIx* = myosin heavy chain IIx; *MyHC IIb* = myosin heavy chain IIb; *FASN* = fatty acid synthase; *PPARα* = peroxisome proliferator-activated receptor α; *CD36* = cluster of differentiation 36/fatty acid translocase; *SCD* = stearoyl-CoA desaturase; *ACC* = acetyl-CoA carboxylase; *PPARG* = peroxisome proliferator-activated receptor γ; *SREBP1* = sterol regulatory element-binding protein 1; *CPT1* = carnitine palmitoyltransferase 1; *FATP1* = fatty acid transport protein 1.

**Table 3:** Effect of FRSM replacing soybean meal on growth performance of finishing pigs.

Items	CSD	FRSM50	FRSM75	FRSM100	SEM	*p*-Value
Initial weight, kg	61.60	61.00	60.43	60.64	1.08	0.985
Final weight, kg	101.84	101.06	101.91	99.14	1.43	0.905
ADFI, g/d	2669.52	2600.64	2648.89	2517.46	46.61	0.688
ADG, g/d	894.29	890.16	921.90	855.56	16.36	0.581
F:G	3.01	2.92	2.89	2.95	0.05	0.896

Note: ADFI = average daily feed intake; ADG = average daily gain; F:G = feed-to-gain ratio.

**Table 4:** Effect of FRSM replacing soybean meal on carcass traits of finishing pigs.

Items	CSD	FRSM50	FRSM75	FRSM100	SEM	*p*-Value
Carcass length, cm	97.50	95.71	96.50	95.08	0.83	0.783
Carcass weight, kg	70.50	70.36	71.46	70.03	0.94	0.962
Carcass yield, %	69.25	69.72	70.33	70.62	0.56	0.843
Loin eye area cm^2^	51.04	53.20	55.00	53.61	1.11	0.668
Skin thickness, mm	3.59	3.49	3.13	2.83	0.16	0.328
Suet weight, kg	1.03	1.01	0.93	0.71	0.05	0.063
Average backfat thickness, mm	20.95	16.73	17.08	15.29	0.77	0.064
Cardiac index	0.35	0.38	0.39	0.38	0.01	0.695
Hepatic index	1.49	1.55	1.54	1.58	0.03	0.783
Splenic index	0.15	0.16	0.17	0.15	0.01	0.481
Renal index	0.29	0.31	0.30	0.32	0.01	0.721
Lung index	1.02	0.94	0.96	0.97	0.04	0.876

**Table 5:** Effect of FRSM replacing soybean meal on serum biochemical indicators of finishing pigs.

Items	CSD	FRSM50	FRSM75	FRSM100	SEM	*p*-Value
TP, g/L	40.89	42.73	46.14	44.14	1.31	0.566
ALB, g/L	33.51	33.07	33.37	32.45	0.46	0.866
GLB, g/L	7.38	9.65	12.77	11.68	1.15	0.377
TC, mmol/L	2.96 ^a^	2.65 ^b^	2.63 ^b^	2.59 ^b^	0.06	0.049
HDL-C, mmol/L	1.26	1.15	1.19	1.08	0.03	0.232
LDL-C, mmol/L	1.55 ^a^	1.34 ^ab^	1.27 ^b^	1.32 ^b^	0.04	0.049
TG, mmol/L	0.762	0.740	0.728	0.663	0.02	0.380
UREA, mmol/L	4.96 ^a^	5.13 ^a^	4.46 ^a^	3.15 ^b^	0.20	<0.001
GLU, mmol/L	4.379	4.619	4.910	4.412	0.15	0.597
AST, U/L	40.03	41.30	46.14	40.20	1.40	0.371
ALT, U/L	67.79 ^a^	59.40 ^ab^	52.40 ^b^	51.49 ^b^	2.37	0.041

Note: ^a, b^ Within a row, mean values with different superscripts differ significantly at *p* < 0.05. n = 7.

**Table 6:** Effect of FRSM replacing soybean meal on the meat quality of finishing pigs.

Items	CSD	FRSM50	FRSM75	FRSM100	SEM	*p*-Value
pH_45min_	6.14	6.03	6.11	6.09	0.03	0.562
L*_45min_	38.20	38.14	38.24	38.47	0.35	0.990
a*_45min_	5.12	5.07	5.20	5.29	0.14	0.953
b*_45min_	1.69	1.60	1.42	1.50	0.05	0.228
pH_24h_	5.44 ^b^	5.42 ^b^	5.43 ^b^	5.48 ^a^	0.01	0.019
L*_24h_	46.64	47.94	48.11	48.72	0.37	0.268
a*_24h_	5.15	5.47	5.62	5.98	0.12	0.068
b*_24h_	2.15	2.13	2.06	2.01	0.08	0.927
Drip loss, %	2.61 ^a^	2.30 ^ab^	2.34 ^ab^	1.85 ^b^	0.10	0.040
Cocking loss, %	26.12	26.84	25.48	24.44	0.62	0.575
Shear force, N	39.55 ^a^	33.59 ^ab^	30.04 ^b^	29.19 ^b^	1.33	0.022
Moisture, %	27.74	28.04	28.01	27.79	0.23	0.963
IMF, %	4.19	5.02	5.31	5.62	0.19	0.075
TG, mmol/gprot	0.12 ^b^	0.15 ^ab^	0.20 ^a^	0.19 ^a^	0.01	0.039
TC, mmol/gprot	0.038	0.038	0.040	0.045	0.002	0.811

Note: ^a, b^ Within a row, mean values with different superscripts differ significantly at *p* < 0.05. n = 7.

**Table 7:** Effect of FRSM replacing soybean meal on muscle texture quality of finishing pigs.

Items	CSD	FRSM50	FRSM75	FRSM100	SEM	*p*-Value
Hardness, g	641.71	595.07	570.57	424.50	45.79	0.387
Chewiness, mJ	13.83 ^a^	9.88 ^ab^	7.98 ^b^	6.47 ^b^	0.98	0.023
Gumminess, g	489.57	401	442.79	353.57	40.43	0.695
Cohesiveness	0.74	0.75	0.75	0.74	0.01	0.395
Springiness, mm	2.99	3.07	3.06	3.11	0.02	0.214

Note: ^a, b^ Within a row, mean values with different superscripts differ significantly at *p* < 0.05. n = 7.

**Table 8:** Effect of FRSM replacing soybean meal on free amino acid contents in LTL of finishing pigs (μg/g).

Items	CSD	FRSM50	FRSM75	FRSM100	SEM	*p*-Value
Histidine	256.41	262.94	259.97	226.75	5.99	0.140
Alanine	72.28	75.87	71.19	75.47	2.45	0.891
Arginine	512.69	612.04	585.85	547.09	16.60	0.146
Serine	7.70	9.96	8.97	8.96	0.32	0.087
Glutamic acid	79.40	99.62	102.07	133.20	7.89	0.097
Aspartic acid	302.51	321.91	343.80	358.94	8.35	0.084
Threonine	20.26 ^b^	28.86 ^a^	31.09 ^a^	29.54 ^a^	1.37	0.012
Lysine	590.23	696.71	703.03	704.06	23.46	0.236
Tryptophan	496.79	512.97	555.20	579.39	17.92	0.369
Tyrosine	246.94	232.94	217.65	206.72	6.35	0.127
Valine	131.17 ^c^	156.59 ^b^	168.65 ^ab^	181.86 ^a^	4.40	<0.001
Methionine	110.22 ^c^	160.05 ^bc^	207.36 ^ab^	262.37 ^a^	14.06	<0.001
Proline	281.75	266.41	238.33	289.54	11.57	0.437
Glycine	3.86	4.34	3.65	3.76	0.14	0.306
Phenylalanine	557.57	486.70	505.25	537.16	14.71	0.324
Isoleucine	444.82 ^b^	460.34 ^b^	474.85 ^b^	536.51 ^a^	10.24	0.006
Leucine	416.35 ^b^	437.68 ^b^	452.85 ^b^	510.26 ^a^	9.74	0.002
Glutamine	152.49	191.46	196.24	251.27	14.83	0.145
Asparagine	33.61 ^b^	35.39 ^b^	36.71 ^b^	42.80 ^a^	1.09	0.015
Cysteine	29.87	27.41	25.80	27.74	0.66	0.166
EAA	3023.82 ^b^	3202.84 ^b^	3358.24 ^ab^	3567.89 ^a^	66.33	0.022
NEAA	1723.11	1877.34	1830.26	1945.49	37.21	0.204
Umami AA	381.92 ^b^	421.53 ^b^	445.87 ^ab^	492.14 ^a^	12.90	0.016
SAA	1128.56	1273.60	1252.49	1362.60	39.83	0.239
BAA	3172.96	3322.24	3427.62	3588.10	61.59	0.114
TAA	4746.92 ^b^	5080.18 ^ab^	5188.50 ^a^	5513.39 ^a^	85.41	0.011
EAA/TAA	0.64	0.63	0.65	0.65	0.01	0.704

Note: Essential amino acids (EAAs) = the sum of valine, isoleucine, leucine, phenylalanine, methionine, tryptophan, threonine, tryptophan, and lysine; Non-essential amino acids (NEAAs) = the sum of alanine, glutamine, arginine, serine, glutamic acid, aspartic acid, tyrosine, proline, glycine, cysteine, and asparagine; Umami amino acids = the sum of glutamic acid and aspartic acid; Sweet amino acids (SAAs) = the sum of alanine, glycine, threonine, serine, proline, glutamine, and lysine; Bitter amino acids (BAAs) = the sum of isoleucine, leucine, phenylalanine, arginine, methionine, valine, histidine, tyrosine, and tryptophan; Total amino acids (TAAs) = total amino acids; ^a, b, c^ Within a row, mean values with different superscripts differ significantly at *p* < 0.05. n = 7.

**Table 9:** Effect of FRSM replacing soybean meal on the fatty acid composition of the LTL in finishing pigs (μg/g).

Items	CSD	FRSM50	FRSM75	FRSM100	SEM	*p*-Value
C11:0	2.44	2.41	2.42	2.76	0.06	0.154
C13:0	2.40	2.57	2.51	3.18	0.17	0.375
C14:0	62.25	61.42	67.35	77.92	2.49	0.062
C14:1	3.12	3.07	3.28	3.69	0.11	0.169
C15:0	18.52	16.07	19.28	22.64	0.92	0.079
C15:1	3.57	3.59	3.11	3.71	0.17	0.639
C16:0	330.77	337.30	324.16	340.08	7.56	0.894
C16:1	44.65	48.20	50.64	55.23	2.52	0.531
C16:2	4.39 ^b^	5.43 ^ab^	5.00 ^b^	6.66 ^a^	0.29	0.032
C18:0	403.39 ^a^	360.68 ^b^	382.74 ^ab^	413.83 ^a^	7.05	0.029
C18:1N9C	156.04	161.49	167.77	180.06	4.53	0.281
C18:2N6	83.46	107.89	107.36	111.22	6.36	0.403
C18:3N3	45.89	59.78	59.94	62.24	2.75	0.132
C18:3N6	45.34	58.47	58.78	60.62	2.65	0.147
C18:4	0.73 ^b^	0.93 ^a^	0.90 ^a^	0.96 ^a^	0.03	0.046
C18:5	71.80 ^c^	73.94 ^b^	74.53 ^ab^	75.87 ^a^	0.40	0.001
C20:0	21.51 ^ab^	18.71 ^b^	19.80 ^b^	22.95 ^a^	0.55	0.023
C20:1	10.01 ^b^	11.77 ^ab^	12.67 ^a^	14.04 ^a^	0.46	0.009
C20:3N6	99.32	121.16	117.05	129.19	5.11	0.208
C20:5N3	3.23	3.28	3.89	5.33	0.35	0.112
C21:0	9.11	7.14	8.74	6.16	0.88	0.628
C21:1	2.99	4.42	4.06	5.00	0.32	0.150
C21:2	1.46 ^b^	1.41 ^b^	1.82 ^ab^	2.29 ^a^	0.11	0.006
C21:5	1.16 ^b^	1.22 ^b^	1.49 ^a^	1.61 ^a^	0.05	<0.001
C22:0	195.67	193.31	184.41	196.83	4.98	0.829
C22:1N9	38.62 ^c^	43.24 ^bc^	47.68 ^b^	53.65 ^a^	1.40	<0.001
C22:5N3	3.62	4.62	4.73	5.03	0.30	0.396
C22:5N6	3.56	4.27	4.49	4.90	0.29	0.435
C22:6N3	1.38	1.67	1.68	1.64	0.13	0.856
SFA	1046.06	999.62	1011.42	1086.36	17.87	0.324
MUFA	259.01	275.78	289.18	315.39	8.27	0.093
PUFA	365.34	444.09	441.67	467.55	15.31	0.089
UFA	624.35	719.87	730.86	782.94	22.76	0.088
n-3 PUFA	54.11	69.35	70.24	74.23	3.07	0.093
n-6 PUFA	231.68	291.79	287.69	305.93	12.12	0.137
n-6/n-3 PUFA	4.33	4.21	4.14	4.15	0.07	0.786
SFA/UFA	1.70 ^a^	1.40 ^b^	1.41 ^b^	1.40 ^b^	0.04	0.004
PUFA/SFA	0.35 ^b^	0.44 ^a^	0.43 ^a^	0.43 ^a^	0.01	0.008

Note: Saturated fatty acids (SFAs) = the sum of C11:0, C13:0, C14:0, C15:0, C16:0, C18:0, C20:0, C21:0, and C22:0; Monounsaturated fatty acids (MUFAs) = the sum of C14:1, C15:1, C16:1, C18:1N9C, C20:1, C21:1 and C22:1N9; Polyunsaturated fatty acids (PUFAs) = the sum of C16:2, C18:2N6, C18:3N3, C18:3N6, C18:4, C18:5, C20:3N6, C20:5N3, C21:2, C21:5, C22:5N3, C22:5N6, and C22:6N3; Unsaturated fatty acids (UFAs) = MUFA + PUFA; n-3 PUFA = the sum of C18:3N3, C20:5N3, C22:5N3, and C22:6N3; n-6 PUFA = the sum of C18:2N6, C18:3N6, C20:3N6, and C22:5N6; ^a, b, c^ Within a row, mean values with different superscripts differ significantly at *p* < 0.05. n = 7.

## Data Availability

The original contributions presented in this study are included in the article/Supplementary Material. Further inquiries can be directed to the corresponding authors.

## Supplementary Materials

The following supporting information can be downloaded at: https://www.mdpi.com/article/10.3390/foods15030587/s1, Table S1: Chemical composition of rapeseed meal mixture before and after fermentation (dry matter basis).
