# Supplementary material for: Improving Meat Quality and Lipid Metabolism of Finishing Pigs by Replacing Dietary Soybean Meal with Enzyme–Bacteria Co-Fermented Rapeseed Meal
Source: Foods. 2026 Feb 6;15(3):587. doi: 10.3390/foods15030587 (PMC12896769; doi:10.3390/foods15030587)
Supplement: Supplementary file 1 [file foods-15-00587-s001.zip › foods-4116401-supplementary.pdf]

Supplementary Materials

Table S1 Chemical composition of rapeseed meal mixture before and after fermentation (dry matter basis)

Ingredient	RSM	FRSM
CP,%	41.59	43.06
EE,%	2.58	3.32
TCA-SP,%	2.10	20.40
Small peptide,%	5.05	47.38
GLS,μmol/g	21.43	5.76
Total phenol,mg/g	9.53	15.33
Flavonoids,mg/g	11.20	15.50

Note: RSM = rapeseed meal mixture; FRSM = fermented rapeseed meal; CP = crude protein; EE = ether extract; TCA-SP = trichloroacetic acid soluble protein; GLS = glucosinolates.
